# Evidence for a curvilinear relationship between posttraumatic growth and posttrauma depression and PTSD in assault survivors

**DOI:** 10.1002/jts.20378

**Published:** 2009-02

**Authors:** Birgit Kleim, Anke Ehlers

**Affiliations:** Department of Psychology, Institute of Psychiatry, King's College LondonLondon, UK

## Abstract

Two studies of assault survivors (Ns = 180, 70) examined associations between posttraumatic growth (PTG) and posttrauma psychopathology. Both studies found significant curvilinear associations between PTG and posttraumatic stress disorder, whereas only Study 1 found a curvilinear association between PTG and depression symptom severity. Survivors with no or high growth levels reported fewer symptoms than those who reported moderate growth. Study 1 also investigated potential PTG predictors. Non-Caucasian ethnicity, religiousness, peritraumatic fear, shame, and ruminative thinking style, assessed at 2 weeks, predicted growth at 6 months. Posttraumatic growth may thus be most relevant in trauma survivors who attach enduring significance to the trauma for their lives and show initial distress. Moderate levels of PTG do not seem to ameliorate posttrauma psychopathology.

Survivors of traumatic events may not only develop psychological problems such as posttraumatic stress disorder (PTSD), but also report positive changes in their lives as a consequence of the trauma (e.g., Frazier, Conlon, & Glaser, 2001; Frazier, Tashiro, Berman, Steger, & Long, 2004; Tedeschi & Calhoun, 2004). These include a feeling of strength, becoming closer to family and friends, or a greater appreciation of life. Such perceived positive changes are summarized in the concept of posttraumatic growth (PTG), and have been observed after a range of traumatic events, such as traumatic bereavement (Davis, Nolen-Hoeksema, & Larson, 1998), combat (Schnurr, Rosenberg, & Friedman, 1993), or man-made disasters (McMillen, Smith, & Fisher, 1997).

Although diverse, all of these traumatic events are uncontrollable, highly threatening, and have potentially irreversible consequences. Intentional acts of violence may have especially profound effects on survivors' posttrauma adaptation (Kessler, Sonnega, Bromet, Hughes, & Nelson, 1995; Tedeschi, 1999), including raised fear, alienation, depression, and posttraumatic stress symptoms (Norris, Kaniasty, & Thompson, 1997). Following an assault, survivors thus commonly report an increased sense of vulnerability and helplessness. At the same time, however, they may also report an increased sense of their own capacities in surviving and overcoming the event (Calhoun & Tedeschi, 2004). Paradoxically, the shattering of previous beliefs can thus be seen as a starting point for posttraumatic growth in that it promotes the development of new perspectives, and a sense that valuable lessons have been learned (Calhoun & Tedeschi, 2006). Although such reports of growth are common across various samples of trauma survivors, it is still unclear whether reports of increased growth do indeed indicate measurable psychological benefits, and what the precise nature of the relationship between growth and psychopathology is. On the one hand, common sense suggests that growth should be related to positive outcome. Indeed, a number of studies found that growth is related to more well-being and less distress, such as to increased personal resources following trauma (Park & Fenster, 2004), more self-esteem (e.g., McMillen et al., 1995), more positive mood (e.g., Carver & Antoni, 2004), as well as less anxiety and depression (Park & Fenster, 2004). On the other hand, however, there is recent evidence that greater levels of growth may also be related to negative affect, more distress over time, and poorer quality of life (Tomich & Helgeson, 2004). Butler et al. (2005) found that growth reported in the first few months after the September 11 terrorist attacks was associated with higher trauma symptom levels. A third group of researchers failed to find significant associations between growth outcome and psychological adjustment (e.g., Cordova, Cunningham, Carlson, & Andrykowski, 2001; Lehman et al., 1993; Grubaugh & Resick, 2007). In linewith these inconsistent results, a recentmeta-analysis indicated that the relationship of growth to psychopathology may be more complicated than previously thought (Helgesson, Reynolds, & Tomich, 2006). A possible explanation for the pattern of results is the recent suggestion that the relationship between growth and distress may not be strictly linear, as most previous studies assumed, but curvilinear (Lechner, Antoni, & Carver, 2006). Some trauma survivors may simply fail to perceive the event as a crisis, and would therefore have little reason for either distress or growth. A second group may experience mostly distress and less growth, and a third group may experience mostly growth and less distress. Combining these three groups in one sample would lead to a curvilinear relation between growth and distress, with the curve traveling from low levels of distress and low growth, through a group with some growth and mostly distress, to those with high levels of growth and lower distress. Data from two samples of women with breast cancer confirmed this pattern (Lechner et al., 2006). A curvilinear association between growth and distress/psychopathology may help resolve discrepancies among studies because the relationships found may depend on what range of perceived growth (and thus which “regions of the curve”) a studied population occupies. To our knowledge, there is only one other study (Butler et al., 2005) that tested for such a nonlinear relation, and found quadratic relationships between PTSD symptoms and growth in a large convenience sample following September 11. It would be important to examine whether such a curvilinear relationship can be replicated in other samples of trauma survivors.

A related question is whether and how different levels of growth can be predicted. Is growth also related to initial candidate predictors in a curvilinear way? Lechner and colleagues (2006) divided the range of growth scores into three segments and found that initial optimism, positive reframing, religious coping, acceptance, and concerns about physical integrity predicted these categories of growth in their sample. Other variables that could predict growth levels can be derived from theoretical and empirical work. A recent theoretical model explained positive changes after trauma as the result of a number of factors, such as characteristics of the person and of the challenging situation, management of emotional distress, and rumination (Calhoun & Tedeschi, 2006). These authors argued that the more an individual needs to work through a traumatic event and its aftermath, themore he or she will subsequently benefit from the experience (Tedeschi & Calhoun, 2004). There is some empirical evidence for the factors proposed by this model, and for some additional factors. First, religiousness has been identified as a fairly consistent predictor of PTG (e.g., Calhoun, Cann, Tedeschi, & McMillan, 2000; Shaw, Joseph, & Linley, 2005 for a recent review). Ethnicity also seems to play a role. African American sexual assault survivors (Kennedy, Davis, & Taylor, 1998) and African American HIV patients (Milam, 2004) reported more posttraumatic positive change than White Americans. A recent meta-analysis also found that ethnic minorities are more likely to report growth, and associations between growth and well-being have been greatest in studies that consisted of a larger percentage of ethnic minority participants (Helgeson et al., 2006). Second, the subjective severity of the trauma, such as perceptions of impending death or injury during the event and degree of initial distress, has been shown to predict PTG (e.g., Frazier et al., 2001). Third, there is also some evidence on the role of posttraumatic processes in the emergence of PTG. Initial, event-related rumination after the trauma, as highlighted in Tedeschi and Calhoun's (1999) theoretical model, was related to greater PTG in different groups of trauma survivors (e.g., Calhoun, Cann, Tedeschi, & McMillan, 2000; Park et al., 1996). So far, studies have only tested for linear relationships of these factors with growth. It is conceivable, however, that they are related to growth in a curvilinear way.

The present study investigated the relationship between growth and psychopathology in two samples of assault survivors. Study 1 assessed perceptions of growth in recent assault survivors at 6 months, and tested whether growth was associated with PTSD and depression symptom severity, and whether this relationship is curvilinear. We then tested whether growth at 6 months can be predicted from a number of candidate demographic variables, peritraumatic emotion, and ruminative thinking style measured at 2 weeks after the assault, and whether these relationships are curvilinear. Study 2 assessed growth in a second sample of assault survivors, who had been assaulted between 3 months and 6 years prior to participating to replicate PTG distributions and potential curvilinearity of the relationship between PTG and PTSD and depression symptom severity.

## METHOD

### Participants

Participants who had experienced an assault were required to meet Criterion A of the *Diagnostic and Statistical Manual of Mental Disorders, Fourth Edition* (*DSM-IV*; American Psychiatric Association, 1996). Exclusion criteria were (a) occurrence of the assault in the context of ongoing domestic violence, (b) insufficient knowledge of English, (c) complete loss of memory of the assault (e.g., due to head injuries), (d) currently psychotic, (e) age less than 18 years. Demographic characteristics are shown in Table 1. Assaults were mainly physical (99%), including common assault, actual bodily harm, grievous bodily harm; 1% were sexual assaults.

**Table 1:** Sample Characteristics for Both Study Samples (Study 1 and 2)

	Study 1: Prospective study (*N* = 180)		Study 2: Cross-sectional study (*N* = 70)	
Variable	*n*	%	*n*	%
Sex; proportion male	122	67.8	44	62.9
Ethnicity; proportion Caucasian	109	60.6	42	60.0
Socioeconomic status^a^				
Very low income (<$15,000)	76	42.2	29	41.4
Low income ($15,000-25,000)	38	21.1	11	15.7
Moderate income ($25,000-55,000)	32	17.8	15	21.4
High income (over $55,000)	23	12.8	8	11.4
Missing/refused information	11	6.1	7	10.0
Marital status				
Single	117	65.0	44	62.9
Married	36	20.0	6	8.6
Divorced/separated/widowed	23	12.8	20	28.6
Refused information	4	2.2	0	0
Education				
No exams	32	17.8	7	10.0
GCSE/ O-Levels^b^	45	25.0	17	24.3
A Level^c^	24	13.3	10	14.3
Bachelor degree	40	22.2	14	20.0
Postgraduate degree	15	8.3	5	7.1
Other	24	13.3	17	24.3
Employment status				
Employed/studying	118	65.6	48	68.6
Unemployed/retired	58	32.2	22	31.4
Other	4	2.2	0	0
Number of assailants				
1	95	52.8	45	64.0
2 or more	85	47.2	25	36.0
Weapon involved; proportion weapon involved	83	46.0	33	47.0
	M	SD	M	SD
Age	35.08	(11.39)	34.77	(11.13)
Days since assault M (SD)	17.53	(7.83)	468.20	(341.07)
PTSD symptom severity (PSSI)^d^ M (SD)	11.31	(10.91)	15.35	(11.21)
Depression symptom severity (BDI)^d^ M (SD)	10.25	(11.55)	14.94	(11.40)

a Combined household income.

b Equivalent to 11 years of education.

c Equivalent to 13 years of education.

d PSSI, BDI (Beck Depression Inventory) scores at 6 months for prospective study (Study 1).

Sample 1 comprised 180 assault survivors drawn from a prospective study of assault survivors recruited at 2 weeks after receiving treatment for their injuries at an inner-city Emergency Department (Kleim, Ehlers, & Glucksman, 2007). The original study sample comprised 222 assault survivors, and 180 (81%) of these filled in the Posttraumatic Growth Inventory (PTGI) and symptom measures 6 months later. Participants who did not complete the PTGI did not differ from the remaining sample in terms of age, sex, ethnicity or PTSD and depressive symptom severity (all *ps* > .06), but were more likely to be religious, *F* (1, 214) = 4.20, *p* < .05.

Sample 2 comprised 70 assault survivors who had been treated for their injuries in the same emergency department 3 to 15 months prior to the study, and participants who had replied to a flyer at a local newsagent. The mean time that had elapsed since the trauma was 39 months (*SD* = 28 months).

### Measures

The General Information Questionnaire was adapted from Halligan, Michael, Clark, and Ehlers (2003) to assess demographic characteristics (age, sex, ethnic background, religion, marital status, income, education).

The Posttraumatic Growth Inventory was developed by Tedeschi and Calhoun (1996) to assess positive changes experienced after trauma. The scale consists of five subscales comprised of 21 items: personal strength, new possibilities, relating to others, appreciation of life, and spiritual change. Items are rated on a 6-point Likert scale, ranging from 0 (I did not experience this change as a result of the assault) to 5 (I experienced this change to a very great degree as a result of the assault). Internal consistency of mean PTGI scores was very good in both samples (21 items, α=.96, Study 1; α=.96, Study 2).

At 6 months, participants were interviewed with the PTSD module of the Structured Clinical Interview for DSM-IV (SCID; First, Spitzer, Gibbon, & Williams, 1996) to determine whether or not they met *DSM-IV* criteria for chronic PTSD, depression, or phobia. Interrater reliability was high (κ = 1 for depression, κ =.82 for PTSD; based on 56 interviews, two raters). The PTSD symptom severity was assessed with the PTSD Symptom Scale-InterviewVersion (PSS-I; Foa, Riggs, Dancu,&Rothbaum, 1993). The interviewer rated each of the PTSD symptoms on a scale from 0 (*not at all*) to 3 (*5 or more times per week/very much*). The total PSS-I score is the sum of the ratings for the 17 items.

Depression symptom severity was assessed with the Beck Depression Inventory (BDI; Beck & Steer, 1987), a widely used, standardized 21-item questionnaire measure of depression of established reliability and validity. Internal consistency in the present sample was excellent (α = .94, for BDI at 6 months in Study 1, α=.92, in Study 2).

We used the fear/shock and shame/humiliation subscales of the Peritraumatic Emotions Questionnaire, the 22-item self-report questionnaire was adapted from Halligan, Michael, Clark, and Ehlers (2003). Participants indicated on a scale from 0 (not at all) to 4 (very strongly) how much they experienced each emotion (e.g., humiliated, shocked, guilty) during the assault and until help arrived. The fear/shock factor comprised the items terrified, frightened, horrified, fearful, helpless, alarmed, shocked, hopeless, frozen, and upset (10 items, α=.90, Study 1). The shame/humiliation subscale included the items ashamed, humiliated, insulted, guilty (four items, α=.71, Study 1).

The Response Styles Questionnaire (RSQ; Nolen-Hoeksema, 1991; Nolen-Hoeksema, Morrow, & Fredrickson, 1993) assesses the frequency with which individuals think about their symptoms, when they feel sad or depressed on a 4-point scale from 1 (never) to 4 (always). The 10-item short version was used for this study. The mean score showed excellent internal consistency (α=.93, Study 1).

### Procedure

In Study 1, participants attended a research session approximately 2 weeks following the assault. A master's level psychologist administered questionnaires and clinical interviews and participants completed questionnaires measuring demographic variables, peritraumatic emotions, and ruminative thinking. At 6 months, participants filled in the PTGI and BDI and were interviewed with the SCID and PTSD Symptom Scale Interview via telephone by the same interviewer. In Study 2, participants attended a research session on average at 39 months after the assault, where they filled in questionnaires and completed the SCID and PSS-I interviews.

Participants received $97 (Study 1)/$49 (Study 2) as reimbursement for time and travel expenses. Differences in reimbursement are due to the longitudinal nature of Study 1, where participants completed additional questionnaires and tasks that have been described elsewhere (e.g., Kleim et al., 2007).

### Data Analysis

The Statistical Package for the Social Sciences (SPSS version 15.0) was used for the analyses. Linear and quadratic relations between growth and symptom severity were tested in both samples. The PTGI scores were first mean-centered and then squared to create the quadratic growth term. In hierarchical regressions, PSSI and BDI scores were then regressed onto the linear PTGI effect in Step 1, and the quadratic PTGI effect in Step 2. To investigate the nature of the relationship between continuous predictor variables at 2 weeks and growth at 6 months in Study 1, we tested for linear and curvilinear relationships in the same way. The influence of dichotomous demographic variables on PTGI scores at 6 months was tested with t tests, i.e., religiousness, sex, and ethnicity. Alpha for all analyses was set to p <.05 (two-tailed).

## RESULTS

### Study 1

Fifty-eight percent of the participants (*n* = 105) reported at least some degree of positive change at 6 months postassault, as reflected by a mean item score above 1 on the 6-point scale PTGI. Posttraumatic growth scores were generally low, with a mean PTGI total score of *M* = 1.59, *SD* = 1.27, range=0–4.64. The highest scores were found for increased appreciation of life (*M* = 1.96, *SD* = 1.47) and perceived personal strength (*M* = 1.73, *SD* = 1.39). Higher PTGI levels were associated with greater PTSD and depression symptom severity, *r* = .43, *p* <.01 for PSS-I; *r* =.35, *p* <.01 for BDI.

**Figure 1:**
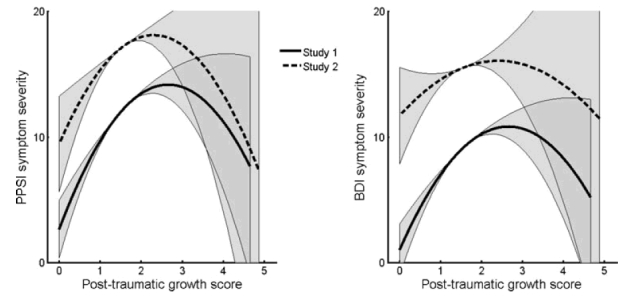
Quadratic relation of posttraumatic growth to PTSD symptom severity (PTSD symptom severity Inventory [PSSI], left figure) and to depression symptom severity (Beck Depression Inventory [BDI], right figure) at 6 months postassault. Gray-shaded areas represent 95% confidence intervals for curvilinear betas; all quadratic relationships were significant, with the exception of the relationship between posttraumatic growth and the BDI in Study 2.

Hierarchical regression examined whether the quadratic component of the relationship between growth and symptoms of PTSD and depression predicts over and above the linear effect. There was a significant quadratic effect of PTGI scores in the prediction of both PTSD symptom severity (PSS-I), β = −.27Δ*R*^2^ =.06, *p* < .001, and of depressive symptom severity (BDI), β=−.24, Δ*R*^2^ =.05, *p* <.01. The negative signs for both quadratic terms' partial correlations indicated that the shape of the relation was such that moderate growth was associated with high symptom severity, whereas low and high growth were related to lower self-reported PTSD and depressive symptoms. Quadratic terms were significant (*p* <.05) for most PTGI subscales. Exceptions were the appreciation of life subscale, where the quadratic terms were only marginally significant for the prediction of PTSD, β=−.14, *p* =.057, relating to others, where the quadratic term was marginally significant for depression, β=−.15, *p* =.08, and spiritual change, where the quadratic term did not predict PTSD symptom severities, β = −.02, *p* =.238. Figure 1 displays the quadratic relations between posttraumatic growth (mean PTGI score) and PTSD and depression symptom severities. The exclusion of the small number of sexual assault survivors did not change these results.

Religious participants reported more growth at 6 months than participants not observing any religion, *t* (172)=4.11, *p* <.001, and non-Caucasian participants reported more growth than Caucasians, *t*(178)=5.78, *p* <.001. Male and female participants did not differ in their mean PTGI scores at 6 months, *t* < 1. The hierarchical regressions for continuous variables showed significant linear relationships between PTGI scores and peritraumatic fear/shock, β =.24, *p* <.001; shame, β=.22, *p* <.01, and ruminative thinking style, β=.18, *p* <.05.None of the quadratic terms were significant, all *ps* > .09.

### Study 2

Sixty percent (*n* = 42) reported at least some degree of positive change since the assault. Posttraumatic growth scores were low, with a mean PTGI total score of 1.56 (*SD* = 1.21, range = 0–4.86). The highest scores were found for an increased appreciation of life (*M* = 2.09, *SD* = 1.47) and perceived personal strength (*M* = 1.70, *SD* = 1.41). Higher levels of perceived growth were associated with greater self-reported PTSD symptom severity, *r* =.53, *p* <.05, but unrelated to depressive symptom severity, *r* =.02, *ns*. Elapsed time since assault was not significantly related to PTG total scores, *r* =.17, *ns*.

For PTSD severity, the results of the hierarchical regression indicated a significant contribution of the quadratic term over and above the linear term, β = −.30, Δ*R*^2^ =.06, *p* <.05. Moderate growth was associated with higher PTSD symptom severity, whereas low and high growth was related to lower PTSD symptoms, as indicated by the negative sign for the quadratic term's partial correlation. In the prediction of depressive symptom severity, neither the linear, nor the quadratic PTGI term were significant, β=−.15, Δ*R*^2^ =.02, *ns*.

## DISCUSSION

Posttraumatic growth researchers have recently raised the question whether “finding something good in the bad is always good?” (Tomich & Helgeson, 2004). Although most studies link posttraumatic growth to positive outcomes, not all research supports the idea that growth is solely beneficial (e.g., Lehman et al., 1993; Park et al., 1996). In line with recent studies (Butler et al., 2005; Lechner et al., 2006), we found evidence for a curvilinear relationship between posttraumatic growth and PTSD and depressive symptom severities in Study 1, and between growth and PTSD in Study 2. Additionally, Study 1 showed that non-Caucasian ethnicity, religiousness, peritraumatic fear, shame, and ruminative thinking style, all assessed at 2 weeks, predicted greater growth at 6 months.

In line with the perceived benefits reported by survivors of sexual assault (Frazier et al., 2004) and survivors of community violence (Updegraff & Marshall, 2005), the present study found that the majority of assault survivors in both studies, almost 60%, reported at least some positive change following their assault.However, growth scores in the present samples were generally lower than in previous studies of trauma survivors (e.g., Cordova et al., 2001; Polatinsky & Esprey, 2000).This may be due to our predominantly male sample; men tend to report fewer benefits than do women (Bates, Trajstman, & Jackson, 2004; Tedeschi & Calhoun, 1996). Furthermore, our sample comprised relatively young, urban assault survivors with predominantly low socioeconomic status and educational levels. This may have contributed to the overall modest level of PTG (see also Cordova et al., 2001; Davis et al., 1998), as substantial levels of PTG may be restricted to those who have more favorable life circumstances contributing to both recovery and growth.

The present project built on recent research suggesting that there are subsets of individuals who report different levels of growth, which are differentially related to posttrauma psychological adjustment (Lechner et al., 2006). In accord with these findings, we found evidence for a curvilinear relationship between posttraumatic growth and PTSD and depressive symptom severities in Study 1, and between growth and PTSD symptom severity in Study 2. Survivors with low or high growth levels reported fewer symptoms than those with intermediate growth levels. The finding that there was a group of trauma survivors who report both low symptoms and low growth is in line with Lechner et al.'s suggestion that some people may fail to regard the trauma as a crisis, accounting for both low symptom and low growth scores. Similarly, authors have stressed that resilient outcomes typically provide little need for PTG (Westphal & Bonanno, 2007). The findings are also consistent with the “shattering of assumptions” hypothesis (Janoff-Bulman, 1992) in that only people who find their previous beliefs shaken by the trauma would be expected to report changes, which may include both negative and positive changes. Thus, only people who attach enduring significance to the trauma, may be motivated to search for new meanings and directions in their life, thus facilitating perceived growth.

The results for the remaining participants appeared to support the beneficial effects of posttraumatic growth in ameliorating the negative effects of trauma. A considerable number of participants showed high levels of posttrauma symptoms and only some growth, whereas a third group experienced mostly growth, and fewer symptoms. Thus, the ability to find benefits and growth following trauma may contribute to better adjustment and less psychopathology.

However, the endorsement of growth cannot be unambiguously interpreted as adaptive. Moderate levels of growth may also reflect trauma survivors' attempts to reassure themselves that the outcome of the trauma is less catastrophic than they think and to minimize symptoms. Maercker and Zoellner (2004) have hypothesized that posttraumatic growth may have an illusory and rather self-deceptive side, possibly linked to denial, avoidance, or wishful thinking. In line with this assumption, Taylor (1983; Taylor & Armor, 1996) found that, in the face of life-threatening illnesses, people often respond with a mildly distorted positive perception of themselves, an exaggerated sense of personal control, and unrealistic optimism (see also Davis & McKearney, 2003). Although Taylor et al. (2000) have related positive distortions of this kind to positive outcome, such reassurance may not always be helpful in overcoming the effects of trauma, which would be consistent with our finding that moderate PTG was associated with persisting psychological problems at 6 months. Moreover, Hobfoll and colleagues (2007) proposed that action is essential to true growth, and that PTG may index positive adaptation when accompanied by actions, not solely by cognitive processes. Although growth action was not measured in the present study, and the active ingredient of such growth action is unknown, it is conceivable that the association between PTG and psychopathology is moderated by growth action. Those who turn growth cognition into action, e.g., by becoming socially or politically active in response to being assaulted, or by actively engaging in getting therapy or socializing with others, may have experienced the protective effect of PTG and showed less psychopathology. To clarify further the role of perceived growth and growth action, more prospective longitudinal studies are needed. Ideally, such studies should assess both cognitive and action-focused growth and psychopathology at several time points posttrauma to understand better the timing of changes in growth and psychopathology.

Whereas both Study 1 and Study 2 found a curvilinear relationship between PTG and PTSD symptom severity, only Study 1 showed a similar pattern for depressive symptoms, and PTG was unrelated to depressive symptoms in Study 2. The latter finding is in accord with nonsignificant cross-sectional associations between growth and depression in samples of survivors of intimate partner violence and earthquakes (Cobb, Tedeschi, Calhoun, & Cann, 2006; Sattler et al., 2006). The main difference between Studies 1 and 2 was that Study 1 recruited participants soon after the trauma and assessed all participants 6 months after the trauma, whereas Study 2 assessed participants on average somewhat later after the trauma, and at more varied intervals. Depression has a fluctuating course, and correlations with other variables may decrease with elapsed time since the trauma. Furthermore, depression levels in Study 2 may have been influenced by further life events and adversity to a larger extent than in Study 1,which also would decrease the importance of the meanings attached to the trauma in this study.

Study 1 also addressed the prediction of posttraumatic growth levels in trauma survivors. Tedeschi and Calhoun (2004) suggested several necessary precursors and PTG predictors: person characteristics, stressor-induced distress, significant challenge to core cognitive schemas regarding the self and the world, and continued cognitive processing. With respect to demographic variables, non-Caucasians reported more growth than Caucasians, and religious people reported more growth than those not observing any religion. This is in line with previous findings (Kennedy, Davis, & Taylor, 1998; Milam, 2004). Further research is needed to identify mediators of these relationships. Greater fear, shame, and humiliation during the assault (reported at 2 weeks) predicted greater growth at 6 months. Similarly, those who engaged more in ruminative thinking at 2 weeks reported greater growth at 6 months than those with lower growth levels. These findings are consistent with the view that initial distress may motivate some people to search for new meanings after trauma and directions in their life, and thus lead to growth.

Although the studies had several strengths including a large sample size and a longitudinal design in Study 1, it also had limitations. First, growth scores were generally low in both samples. It would be desirable to repeat the present analysis in a sample of trauma survivors with higher levels of growth, especially to validate the scores of the subgroup with large growth. The results are limited to survivors of physical assaults, and may not generalize to other traumas. The present study cannot establish causal inference. It remains unclear whether growth is a predisposing factor or a consequence of psychopathology. Distress is likely to set in at an early stage, whereas growth experiences are mostly experienced at a later stage posttrauma, although authors have reported positive changes, such as more empathy, greater appreciation for life, and improvements in relationships, as early as 2 weeks postassault (Frazier et al., 2001). In the aftermath of a traumatic event, psychopathology and growth are likely to influence each other, and may at times evolve independently, possibly at different rates. Future research will need to assess cognitive and action-oriented growth and psychopathology at several time-points posttrauma to answer these questions.

In closing, the present studies add to the current literature on the relationship between growth and posttrauma psychopathology, by showing that growth can be related to PTSD and depression symptoms in a nonlinear way in that both assault survivors with no or high growth levels report fewer symptoms than those with moderate growth. The crucial step will be to disentangle subgroups of trauma survivors or contexts in which PTG is truly endorsed, and potentially put into action, from those endorsing PTG as a somewhat illusionary attempt to reduce cognitive dissonance and lessen the impact of trauma. Such subgroups would call for different types of interventions.
